# Resource utilisation and cost of ambulatory HIV care in a regional HIV centre in Ireland: a micro-costing study

**DOI:** 10.1186/s12913-015-0816-1

**Published:** 2015-04-03

**Authors:** Aline Brennan, Arthur Jackson, Mary Horgan, Colm J Bergin, John P Browne

**Affiliations:** Department of Epidemiology and Public Health, University College Cork, Cork, Ireland; Cork University Hospital and Mercy University Hospital Cork, Cork, Ireland; School of Medicine, University College Cork and Cork University Hospital, Cork, Ireland; St James’s Hospital, Dublin and Trinity College Dublin, Dublin, Ireland

**Keywords:** HIV, Micro-costing, Cost, Healthcare utilisation, Ambulatory care, Outpatient care

## Abstract

**Background:**

It is anticipated that demands on ambulatory HIV services will increase in coming years as a consequence of the increased life expectancy of HIV patients on highly active anti-retroviral therapy (HAART). Accurate cost data are needed to enable evidence based policy decisions be made about new models of service delivery, new technologies and new medications.

**Methods:**

A micro-costing study was carried out in an HIV outpatient clinic in a single regional centre in the south of Ireland. The costs of individual appointment types were estimated based on staff grade and time. Hospital resources used by HIV patients who attended the ambulatory care service in 2012 were identified and extracted from existing hospital systems. Associations between patient characteristics and costs per patient month, in 2012 euros, were examined using univariate and multivariate analyses.

**Results:**

The average cost of providing ambulatory HIV care was found to be €973 (95% confidence interval €938 - €1008) per patient month in 2012. Sensitivity analysis, varying the base-case staff time estimates by 20% and diagnostic testing costs by 60%, estimated the average cost to vary from a low of €927 per patient month to a high of €1019 per patient month. The vast majority of costs were due to the cost of HAART. Women were found to have significantly higher HAART costs per patient month while patients over 50 years of age had significantly lower HAART costs using multivariate analysis.

**Conclusions:**

This study provides the estimated cost of ambulatory care in a regional HIV centre in Ireland. These data are valuable for planning services at a local level, and the identification of patient factors, such as age and gender, associated with resource use is of interest both nationally and internationally for the long-term planning of HIV care provision.

## Background

Highly active anti-retroviral therapy (HAART), available since the mid-1990s, has transformed HIV infection into a chronic disease [1]. Nowadays, with timely diagnosis and engagement in care, people living with HIV can have a life-expectancy that approaches that of the general population [2,3]. However, HAART is not a curative treatment and most newly diagnosed HIV patients face the daunting prospect of decades of medication use and regular healthcare visits. It has also become apparent that long term HIV infection and medication use is associated with increased non-AIDS morbidity [1,4,5].

Since HIV testing began in the early 1980s over 6,600 people have been diagnosed with HIV in Ireland [6]. A prevalence study of patients in care found 3,254 patients accessed HIV care in Ireland in the 12-month period from July 2009 to June 2010, with 25% over 45 years of age [7]. Of these patients, 79% were on treatment and 94% of those had a viral load < 500 copies/ml [7], figures that compare favourably with other developed countries [8-10]. In Ireland, as in many other countries, policy makers and clinicians are now faced with the challenge of continuing to provide high quality care in the context of increasing demand and within on-going financial constraints.

Data on the use and cost of services are needed to estimate the potential impact the changing HIV epidemiology will have on the health service in Ireland, as well as to support evidence based decisions about new models of service delivery, technologies and medications [11]. It is not generally appropriate to extrapolate costs from studies in different countries due to variation in how health services are provided and organised.

There are two general approaches to costing healthcare: top-down and bottom up. A top-down approach estimates the cost of an individual service on average, usually using routinely available data e. g. average *per diem* costs. Top-down costing studies tend to be relatively quick and easy to carry out, however they are also less precise and cannot provide information on individual factors driving the costs [12]. A bottom-up approach (micro-costing) generates a more precise estimate, but is more difficult to perform. In micro-costing all resources used are identified and then the unit costs of the resources are multiplied by the quantities used [12]. Studies examining the differences between the cost estimates produced by the two approaches have concluded that bottom-up approaches are preferable for estimating cost components which have a large impact on total costs (e.g. labour, drugs), for services where there is wide variation in costs between patients and for centres which are integrated within a larger hospital compared to stand-alone centres [13-15].

Existing data on the cost of outpatient care in Ireland are limited. The national average cost of an outpatient visit in Ireland was estimated to be €130 in 2011 using a top-down methodology (National Casemix Programme, date of communication 29/09/2013), however no information is available on how this cost may vary across specialties. A previous study looking specifically at the pharmacoeconomics of HIV in Ireland carried out in the early HAART era reported an average outpatient cost of IR£493 per active patient month in 1999 which would be equivalent to €1,184 in 2012 (inflated using the Irish consumer price index for health) [16,17].

Ireland is currently in the process of restructuring its healthcare funding system from one where hospitals are funded based on historical levels of funding adjusted for activity and patient mix to a prospective case based payment system (“Money Follows the Patient”) [18]. While it is initially planned to implement this change for in-patient and day-case activity, the new funding system will also encompass outpatient services [18]. It is proposed that prices in the new system will be set initially with reference to average prices, but with a view to implementing best practice prices on an incremental basis [18].

The aim of this study was to estimate the total cost of providing an outpatient HIV service in a single centre in 2012 from the health service provider perspective, and to identify associated patient factors. We also compare our results with other available sources of cost data.

## Methods

### Ethics

Ethical approval for this study was obtained from the Clinical Research Ethics Committee of Cork University Hospital.

### Description of service

Cork University hospital (CUH) is one of the largest university teaching hospitals in Ireland, and provides HIV care for the counties of Cork, Kerry, Waterford, and South Tipperary (covering approximately 20% of the Irish population). The infectious disease (ID) department holds two routine outpatient clinics specifically for HIV patients: the main HIV clinic (“HIV clinic”) held once a week and a joint ID-maternity clinic (“antenatal-HIV clinic”) held once a month in the adjoining maternity hospital. Patients attending the antenatal-HIV clinic attend both obstetric and ID services during their appointment.

Local clinical guidelines, based on international best-practice [19-21], recommend that a CD4 count, viral load (VL), full blood count (FBC) and biochemistry patient profile (renal, liver and bone profiles) are performed every 3–4 months. Additional and/or more frequent testing is recommended at initiation of care and at initiation of HAART until suppression is achieved. Antenatal HIV patients are monitored on a monthly basis.

### Study participants

All patients who attended for HIV care in 2012 were identified from a pre-existing clinical database which contains the results of all viral load tests performed in CUH. The database also contains basic demographic and risk factor information on the patients who have attended the public HIV clinic in CUH. Patients who attended the public HIV clinic at least once in 2012 were eligible to be included in the study. Patients who attended for private HIV outpatient care, who did not attend any outpatient appointment in 2012, or who routinely attended another HIV centre for their HIV care were excluded.

For new patients to the clinic in 2012 (newly diagnosed or transferred from another clinic) the date of their first appointment attended or admission was taken as the start date of their care, for all other patients the start date of care was 01/01/2012. Patients were considered to be in care until 31/12/2012 if they used any hospital service (outpatient/in-patient/emergency department) at least once between 01/07/2012 and 31/12/2012. The files of patients who did not use any hospital service in the last 6 months of 2012 were manually checked, and these patients were censored at the date of their last scheduled appointment where appropriate. Patients who had either a CD4 count < 350 cells/μl or an AIDS defining illness at the time of diagnosis recorded in their medical notes were categorised as late diagnoses.

### Resource use

Information on the number and dates of all outpatient appointments and diagnostic tests (laboratory, radiology and cardiology) were extracted from the hospital information system. Data on the total number of months of each HAART regimen dispensed, as well as any relevant start and/or stop dates, were extracted from pharmacy patient files. The medical records of patients with no pharmacy files were cross-checked to confirm the patient did not receive HAART in 2012. In addition, information on the number of doses of prophylactic antibiotics (azithromycin, dapsone, sulfamethoxazole/trimethoprim and pyrimethamine) dispensed to HIV patients was provided by the hospital pharmacy. Diagnostic tests were linked with patient appointments on the basis of matching test and appointment dates. Radiology and cardiology tests, which may not occur on the date of the outpatient appointment where the test was ordered, were linked with the nearest previous outpatient appointment attended.

### Costs

A micro-costing study was carried out to estimate the cost of typical HIV outpatient appointments from the service-provider perspective in 2012. Staff routinely involved in providing or supporting the HIV clinic (clerical, nursing, medical, phlebotomy, and pharmacy) were interviewed to assess the HIV-clinic related tasks performed by the staff member and the estimated patient-facing and non patient-facing time involved. Doctors and nurses self-recorded their patient-facing times at six consecutive HIV clinics in March and April 2013 (127/153 appointments timed). Pharmacy staff self-recorded their HIV related patient-facing and non patient-facing time for the month of April, 2013. The accuracy of self-timed doctor patient-facing times was confirmed by independent observation of approximately 50% of appointments. Average costs per appointment were then calculated based on estimated time and salary of relevant staff using recommended national guidelines [22].

Eight basic types of appointment were identified: 1) did not attend scheduled outpatient appointment (DNA), patient was not on HAART at the time (“DNA, not on HAART”); 2) DNA, patient was on HAART at the time (“DNA, on HAART”); 3) attended the HIV-clinic, patient was not on HAART at the time (“Attended HIV clinic, not on HAART”); 4) attended the HIV-clinic, patient was on HAART at the time (“Attended HIV clinic, on HAART”); 5) attended the antenatal HIV-clinic, patient was not on HAART at the time (“Attended antenatal-HIV clinic, not on HAART”); 6) attended the antenatal-HIV clinic, patient was on HAART at the time (“Attended antenatal-HIV clinic, on HAART”); 7) first attended appointment with the ID service for HIV care, patient was not on HAART at the time (“Baseline visit, not on HAART”); and 8) first attended appointment with the ID service for HIV care, patient was on HAART at the time (“Baseline visit, on HAART”). Appointments were categorised as DNA if the patient did not attend their scheduled appointment without cancelling or rescheduling the appointment prior to the start of the clinic they were due to attend. The cost of a DNA appointment was estimated based on the same non patient-facing administrative and pharmacy staff time (where applicable) as attended appointments as well as the time incurred rescheduling an appointment. In general, a patient is considered lost to follow-up if they DNA three consecutive appointments. For the antenatal-HIV clinic appointments only the cost of the care delivered by the ID team was included in the micro-costing.

### Unit costs

The unit costs of diagnostic tests were obtained from the finance department of the hospital and the National Virus Reference Laboratory. The monthly costs of anti-retroviral medications and prophylactic antibiotics were provided by the hospital pharmacy. In Ireland, the price of all prescribed drugs are set at a national level [23,24], although individual hospitals can negotiate discounts with wholesalers. The number of months of regimens dispensed was used in the calculation of HAART costs to allow for possible wastage due to regimen switching.

The hospital finance department also provided outpatient costs per patient for comparison purposes from a newly implemented activity based costing system. These data were broken down into direct and indirect costs by category (clinical salaries, nursing salaries, non-clinical salaries, imaging, pathology etc.), but contained little information on individual patient characteristics, appointment types or diagnostic tests. Drug costs (with the exception of a few very high cost drugs) were not assigned at a patient level in the hospital activity based costing system in 2012.

### Data analysis

All information was entered into a Microsoft Access ® database. Data were analysed using Microsoft Excel® and STATA 12 (College Station, Texas). Weighted average costs per patient month (ppm) were calculated to account for differences in patient lengths of follow-up. Chi-squared tests were used to test for differences in proportions. Logistic regression was used to estimate the odds ratios of being treated with PI-based regimens compared to NNRTI-based regimens in patients receiving a single type of regimen. Generalized linear models were used to model costs ppm on HAART as they are suitable for modelling cost data, which tend to be skewed, without the need for transformation [25]. The most appropriate family was chosen using the modified Parks test, and the link was chosen using the Pearson Correlation Test, the Pregibon Link Test and the modified Hosmer and Lemeshow test to evaluate power links in increments of 0.1 between 0 and 1 [26].

### Unit costs

The unit costs of individual appointment types, common diagnostic tests and regimen types are shown in Table 1.

**Table 1:** **Unit costs (in 2012 euros) and frequency of appointment types, most common diagnostic tests and most common HAART regimens dispensed**

**Cost category**	**Unit cost**	**Resource use**	**Source of cost data**
*Appointment type*	*Staff cost per appointment*	*Number of appointments*	
DNA, not on HAART	€12.68	32	Micro-costing study
DNA, on HAART	€21.25	127	Micro-costing study
Attended HIV clinic, not on HAART	€174.89	89	Micro-costing study
Attended HIV clinic, on HAART	€194.88	997	Micro-costing study
Attended antenatal-HIV clinic, not on HAART	€174.26	2	Micro-costing study
Attended antenatal-HIV clinic, on HAART	€194.25	42	Micro-costing study
Baseline appointment, not on HAART	€244.46	14	Micro-costing study
Baseline appointment, on HAART	€264.45	12	Micro-costing study
*Most common diagnostic tests*	*Cost per test*	*Number of tests*	
CD4	€47.29	1013	Hospital Finance Dept
Viral load	€92.94	995	National Virus Reference Laboratory
Full Blood Count	€6.79	1036	Hospital Finance Dept
Biochemistry profiles, weighted average cost	€18.63	1042	Hospital Finance Dept
All other tests, weighted average cost	€28.39	1262	Hospital Finance Dept
*HAART regimens*	*Cost per month*	*Number of months dispensed*	
Efavirenz/Emtricitabine/Tenofovir (NNRTI-based regimen)	€816.61	1515	Hospital pharmacy
Emtricitabine/Tenofovir, Darunavir, Ritonavir (PI-based regimen)	€1,097.51	864	Hospital pharmacy
Emtricitabine/Tenofovir, Atazanavir, Ritonavir (PI-based regimen)	€1,084.84	389	Hospital pharmacy
Emtricitabine/Tenofovir, Lopinavir/Ritonavir (PI-based regimen)	€1,148.79	99	Hospital pharmacy
Emtricitabine/Tenofovir, Nevirapine (NNRTI-based regimen)	€747.98	75	Hospital pharmacy
Lamivudine/Zidovudine, Lopinavir/Ritonavir (PI-based regimen)	€953.16	53	Hospital pharmacy
All other regimens, weighted average cost	€923.70	327	Hospital pharmacy
*Prophylactic antibiotics*	*Cost per dose*	*Number of doses dispensed*	
Azithromycin	€0.42	330	Hospital Pharmacy
Sulfamethoxazole and trimethoprim	€0.15	2047	Hospital Pharmacy
Dapsone	€0.69	344	Hospital Pharmacy

DNA, did not attend. “Staff cost” is the estimated total patient and non patient-facing staff costs per appointment. Diagnostic tests includes laboratory, radiology and cardiology tests. “Biochemistry profiles” refers to the number and weighted average cost of the following biochemistry profiles: “patient”, “ward”, “bone”, “renal/bone”, “patient & lipid”, “patient & lipoprotein”, “urea, electrolytes & creatinine” and “liver function tests”.

### Sensitivity analysis

To reflect the uncertainty in the estimates of staff time and laboratory costs staff times were varied by 20% and unit costs of diagnostic testing by 60% in a sensitivity analysis. The 20% variation in staff time was based on the approximate width of the confidence intervals of the average doctor and nurse patient-facing times, while the choice of 60% variation for diagnostic testing costs was based on the difference in the weighted average test costs calculated using alternative unit costs which were available for a limited number of the tests.

## Results

### Study population

In total 326 patients (3659 patient months) were included in this study. The characteristics of the patient population can be seen in Table 2. The majority of patients (59%) were men and the average age of patients was 40 years. Co-infection with hepatitis C virus (HCV) was recorded for 7% of patients, Hepatitis B Virus (HBV) in 2% of patients and both HBV and HCV in 1% of patients. Eight percent (26/326) of patients were new to the service in CUH in 2012, most of whom were newly diagnosed in 2012 (n = 17). The CD4 profile of patients improved with the number of years since diagnosis (Chi-squared test, P = 0.001).

**Table 2:** **Number and proportion of patients and patient-months by patient characteristics**

**Characteristics**		**Patients (%)**	**Patient months (%)**
Patient type	Existing patient	300 (91%)	3506 (95%)
	New patients	26 (8%)	153 (4%)
Gender	Male	192 (59%)	2148 (58%)
	Female	134 (41%)	1511 (41%)
Age	<50 years	284 (87%)	3172 (86%)
	50+ years	42 (13%)	487 (13%)
Risk factor	COHP	133 (41%)	1501 (41%)
	Heterosexual	63 (19%)	721 (20%)
	MSM/Bisexual	95 (29%)	1067 (29%)
	IDU	18 (5%)	185 (5%)
	Other	8 (2%)	96 (3%)
	Unknown	9 (3%)	89 (2%)
Late diagnosis	No	135 (41%)	1535 (42%)
	Unknown	77 (23%)	840 (23%)
	Yes	114 (35%)	1284 (35%)
Years since diagnosis	11+ years ago	95 (29%)	1107 (30%)
	6-10 years ago	107 (33%)	1231 (34%)
	1-5 years ago	100 (30%)	1140 (31%)
	diagnosed in 2012	17 (5%)	105 (3%)
Lowest CD4 count in 2012 (cells/μl)	500+	168 (51%)	1894 (52%)
	350-499	85 (26%)	973 (26%)
	200-349	52 (16%)	581 (16%)
	50-199	14 (4%)	146 (4%)
	<50	7 (2%)	65 (2%)
Treatment and viral load status^b^	On HAART, suppressed	211 (64%)	2469 (67%)
	On HAART, not suppressed	37 (11%)	438 (12%)
	On HAART, unable to determine status	7 (2%)	75 (2%)
	Started/Stopped HAART	49 (15%)	440 (12%)
	Not on HAART	22 (7%)	237 (6%)
**Overall**		**326 (100%)**	**3659(100%)**

COHP, person from a country of high prevalence with no other identifiable risk factor available. IDU, current/former injecting drug user. Late diagnosis defined as CD4 count < 350 cells/μl at diagnosis or AIDS defining illness at diagnosis. “On HAART” means the patient was on HAART for the entire duration of the study, “suppressed” was defined as all viral loads in 2012 < 50 copies/ml, with one test result of 50–499 copies/ml allowed if previous and subsequent viral load results were < 50 copies/ml.

Three patients died in 2012, two of these were considered HIV-related deaths both of which were of people who were newly diagnosed with advanced illness (CD4 < 50 cells/μl) at the time of diagnosis.

### Resource use

There were a total of 1315 HIV outpatient appointments scheduled during 2012, with an average of 4.1 (median 4, range 1–15) scheduled appointments per patient and 3.6 (median 3, range 1–11) attended appointments per patient. The frequencies of each type of appointment are shown in Table 1.

In total 304 patients were on HAART for 3272 patient-months, with 3322 months of HAART dispensed in total. The majority of regimens dispensed consisted of a nucleoside reverse-transcriptase inhibitor (NRTI) backbone combined with either a protease inhibitor (PI) or a non-nucleoside reverse-transcriptase inhibitor (NNRTI). NNRTI-based regimens were the most frequently dispensed (164 patients, 1755 months dispensed) followed by PI-based regimens (145 patients, 1477 months dispensed), with a small number of patients being dispensed other regimens types (10 patients, 90 months dispensed). Fifteen percent (n = 45) of patients had more than one regimen prescribed during the study period, most of which were within the same class, although a small number of patients were prescribed regimens from different classes during the study period (n = 15).

### Total costs

The overall average cost of providing HIV outpatient care was estimated to be €973 ppm in 2012 (median €940, interquartile range €938-€1008). As the cost of HAART accounted for the majority (88%) of total costs, varying the staff time estimates and unit costs of diagnostic testing did not result in substantial changes to the average costs ppm in the sensitivity analysis, with total costs ppm only varying by about 5% (Table 3). When costs were stratified by whether the patient was on HAART or not at the time, the average cost ppm not on HAART was €107 (95% CI €65-€150) compared to €1085 (95% CI €1060-€1111) ppm on HAART.

**Table 3:** **Average base-case cost, in 2012 euros, per patient month (ppm) and results of sensitivity analysis**

	**Base case (95% CI)**	**Lower estimate (95% CI)**	**Upper estimate (95% CI)**
Staff costs ppm (+/− 20%)	€62 (€59-€65)	€50 (€47-€52)	€75 (€71-€78)
Diagnostic test costs ppm (+/−60%)	€55 (€52-€59)	€22 (€21-€23)	€89 (€83-€94)
**Total non-drug cost ppm**	**€117 (€111-€124)**	**€72 (€68-€76)**	€**163 (**€**154-**€**172)**
**Total cost ppm (including drug costs)**	€**973 (**€**938-**€**1,008)**	€**927 (**€**893-**€**962)**	€**1,019 (**€**983-**€**1,054)**

### Patient factors

The mean total cost ppm generally differed little across patient groups (Table 4). However, patients who were on HAART for the duration of their time in the study and were categorised as suppressed were found to have lower average total monthly costs compared to those who were not suppressed (mean €1043 ppm compared to €1189 ppm respectively).

**Table 4:** **Costs per patient month (ppm) by patient subgroup**

	**N**	**Mean drug cost ppm (95% CI)**	**Mean non-drug costs ppm (95% CI)**	**Mean total cost ppm (95% CI)**
**Patient type**				
Existing patient	300	€863 (€829-€897)	€112 (€107-€116)	€975 (€940-€1,010)
New patients	26	€678 (€503-€853)	€253 (€206-€300)	€931 (€732-€1,130)
**Gender**				
Male	192	€844 (€805-€882)	€117 (€109-€124)	€960 (€921-€999)
Female	134	€873 (€812-€934)	€118 (€108-€129)	€991 (€927-€1,055)
**Age group**				
<50 years	284	€859 (€821-€897)	€121 (€114-€128)	€980 (€941-€1,019)
50+ years	42	€833 (€773-€894)	€94 (€85-€104)	€928 (€871-€984)
**Risk factor**				
COHP	133	€878 (€826-€931)	€117 (€106-€127)	€995 (€940-€1,050)
MSM/Bisexual	95	€826 (€769-€884)	€122 (€110-€134)	€948 (€889-€1,007)
Heterosexual	63	€835 (€745-€925)	€113 (€101-€125)	€948 (€856-€1,041)
IDU	18	€931 (€834-€1,027)	€124 (€103-€144)	€1,054 (€942-€1,166)
Other	8	€982 (€733-€1,231)	€92 (€59-€125)	€1,075 (€817-€1,332)
Unknown	9	€697 (€383-€1,012)	€125 (€92-€159)	€823 (€514-€1,132)
**Late diagnosis**				
No	135	€810 (€748-€871)	€120 (€111-€130)	€930 (€867-€993)
Unknown	77	€868 (€797-€939)	€115 (€103-€126)	€983 (€908-€1,058)
Yes	114	€902 (€862-€942)	€116 (€104-€127)	€1,018 (€974-€1,062)
**Years since diagnosis**				
11+ years ago	95	€879 (€830-€929)	€102 (€95-€108)	€981 (€930-€1,032)
6-10 years ago	107	€890 (€833-€947)	€107 (€99-€115)	€997 (€938-€1,056)
1-5 years ago	100	€831 (€762-€899)	€130 (€119-€141)	€961 (€889-€1,033)
diagnosed in 2012	17	€581 (€349-€812)	€275 (€208-€342)	€855 (€582-€1,128)
**Lowest CD4 count in 2012 (cells/μl)**				
500+	168	€888 (€844-€932)	€104 (€98-€110)	€993 (€947-€1,038)
350-499	85	€807 (€734-€879)	€120 (€108-€132)	€927 (€852-€1,002)
200-349	52	€830 (€735-€925)	€129 (€114-€145)	€960 (€861-€1,058)
50-199	14	€839 (€725-€953)	€179 (€115-€244)	€1,018 (€873-€1,164)
<50	7	€903 (€706-€1,100)	€218 (€118-€319)	€1,121 (€842-€1,401)
**Treatment and viral load status**				
On HAART, suppressed	211	€939 (€915-€964)	€104 (€100-€109)	€1,043 (€1,017-€1,069)
On HAART, not suppressed	37	€1035 (€987-€1,083)	€154 (€134-€175)	€1,189 (€1,133-€1,245)
On HAART, undetermined status	7	€914 (€776-€1,052)	€84 (€55-€113)	€998 (€845-€1,151)
Started/Stopped HAART	49	€660 (€578-€742)	€169 (€141-€197)	€829 (€733-€924)
Not on HAART	22	-	€103 (€79-€128)	€103 (€79-€128)
**Overall**	**326**	€**856 (**€**822-**€**889)**	€**117 (**€**111-**€**124)**	€**973 (**€**938-**€**1,008)**

COHP, person from a country of high prevalence with no other identifiable risk factor available. IDU, current/former injecting drug user. Late diagnosis defined as CD4 count < 350 cells/μl at diagnosis or AIDS defining illness at diagnosis. “On HAART” means the patient was on HAART for the entire duration of the study, “suppressed” was defined as all viral loads in 2012 < 50 copies/ml, with one test result of 50–499 copies/ml allowed if previous and subsequent viral load results were < 50 copies/ml.

HAART accounted for the vast majority of outpatient costs. The unit cost of individual regimens varied from a minimum of €552/month to a maximum of €1600/month. Although NNRTI-based regimens tend to be less expensive than PI-based regimens (the weighted average cost of NNRTI-based regimens in this study was €809/month compared to €1091/month for PI-based regimens), there are many other factors apart from cost which influence regimen choice, including patient (e.g. demographic, behavioural), clinical (resistance, adverse reactions, co-morbidities) and health system factors (drugs available for prescription, clinician preferences). Significantly lower proportions of new patients and newly diagnosed patients received HAART in 2012 compared to existing and previously diagnosed patients (Chi-squared test, P = 0.008 and P =0.017, respectively). While a significantly higher proportion of those diagnosed late (i.e. with a CD4 count <350 cells/μl or an AIDS defining illness at diagnosis) received HAART (Chi-squared test, P = 0.003). On multivariate logistic regression of these three factors late diagnosis was the only factor that remained significant with an odds ratio (OR) of 15.5 (95% CI 2–120). Logistic regression of regimen type (analysis restricted to patients only prescribed PI-based regimens or only prescribed NNRTI-based regimens, n = 283) found women were significantly more likely to be prescribed a PI-based regimen (OR 6.6, 95% CI 3.3-13.2, p < 0.001), while those over 50 years were less likely to be prescribed a PI-based regimen (OR 0.34, 95% CI 0.14-0.85, p = 0.022) when adjusted for risk factor, patient type, years since diagnosis, CD4 < 350 cells/μl and late diagnosis.

The results of multivariate analysis of non-HAART, HAART and total costs ppm on HAART are shown in Table 5.

**Table 5:** **Results of multivariate analysis of non-HAART, HAART and total costs ppm on HAART**

	**Non HAART cost ppm on HAART Coefficient** ^**1**^	**HAART cost ppm Coefficient** ^**2**^	**Total cost ppm on HAART Coefficient** ^**2**^
New patient	−6.555*	−0.03	−0.004
Female	1.464	0.138*	0.139*
Age 50+ years	−3.113*	−0.101*	−0.117*
*Risk factor*			
COHP			
MSM	1.308	0.033	0.037
Heterosexual	0.32	0.054	0.054
Other/unknown	−0.737	0.05	0.054
*Late diagnosis*			
No	ref	ref	ref
Unknown	−0.354	−0.012	−0.015
Yes	−0.515	−0.035	−0.04
*Years since diagnosis*			
11+ years ago	ref	ref	ref
6-10 years ago	−0.267	0.053	0.05
1-5 years ago	1.825	0.041	0.059
diagnosed in 2012	4.504	0.078	0.128
Min CD4 < 350 cells/μl	3.432*	0.02	0.054
On HAART, not suppressed/other	2.051	0.059	0.089*
Constant	31.314	3.896	4.026

1) Generalised linear model with Inverse Gaussian family and link power 0.5. 2) Generalised linear model, with Gaussian family and link power 0.2. * p < 0.05. PPM, per patient month. COHP, person from a country of high prevalence with no other identifiable risk factor available. IDU, current/former injecting drug user. Late diagnosis defined as CD4 count < 350 cells/μl at diagnosis or AIDS defining illness at diagnosis. “On HAART” means the patient was on HAART for the entire duration of the study, “suppressed” was defined as all viral loads in 2012 < 50 copies/ml, with one test result of 50–499 copies/ml allowed if previous and subsequent viral load results were < 50 copies/ml. “On HAART, other” includes patients on HAART who were not suppressed or had undetermined status and those who started/stopped HAART in 2012.

### Comparisons of total costs with other sources of cost data

Patient level costs for the HIV patients included in the micro-costing study were provided by the hospital finance department. The data are activity based costing data, the cost components of which are generated using a top-down methodology. The total number of HIV outpatient appointments in the finance dataset was very similar to the total scheduled number of appointments from the utilisation study (1310 vs 1315) which would be expected given that the finance costs are based on information recorded in the same hospital information system as the utilisation data were extracted from. As the costs of most drugs were not assigned at a patient level in this system in 2012, it was not appropriate to compare drug costs, however the estimated non-drug cost ppm based on the hospital activity-based cost data was €63 ppm (95% CI €59-€67) compared to €117 (95% CI €111-€124) in the micro-costing study. Use of the national average outpatient appointment cost (€130.56 in 2012) would have resulted in an even lower estimate of €47 ppm (95% CI €44-€49 ppm). While our estimate is greater than the estimates generated using routine data, the estimated total cost (€973 ppm) is about 20% lower than the IR£493 per active patient month in 1999 reported by a previous micro-costing study (equivalent to €1,184 in 2012). Although that study was carried out in the early HAART era and the patient population was quite different to the patient population in this study [16].

## Discussion

This study reports the results of a bottom-up costing study of routine HIV outpatient care performed in a regional referral centre in the south of Ireland. The total estimated cost of the service in 2012 was €973 ppm, nearly 90% of which was due to the cost of HAART. While it is difficult to compare costs across countries, HAART is reported to account for 60%-80% of the total cost of HIV care (including inpatient care) in many studies in developed countries [27-33].

Despite its cost, HAART is considered a very cost-effective treatment [19,34], and also has the important population-level benefit of reducing onward transmission [35]. HAART also offers the greatest opportunity for reducing costs, for example, in this study the total non-drug spend would have to be reduced by over 35% to save the same amount as could be achieved by a 5% reduction in HAART costs. Possible strategies for reducing HAART costs do exist, such as increased use of generic drugs or less expensive regimens where available and appropriate [36-38], though efforts to reduce drug spend should not come at the cost of increased risk of poorer outcomes and reduced quality of care [19]. As regimen choice is influenced by patient factors, such as age, gender and risk factor, variation in costs between centres at a national level is likely to be a reflection of differences in patient populations, although clinician preference may also play a role. With time, as more patients switch onto newer treatments, the drug costs are likely to increase unless savings can be made through increased use of generic drugs [36-38] or other rationalisation measures. In any case, further research is warranted into the patient and healthcare factors influencing regimen choice, such as the acceptability and potential impact of a preferred medicine scheme.

The most important factor on multivariate analysis which influenced total costs ppm on HAART was gender, with women being 15% more expensive than men. It was also interesting to note that patients over 50 years of age had significantly reduced costs ppm on HAART. This was due to a combination of older patients tending to be on less expensive NNRTI-based regimens, as well as having fewer scheduled appointments (0.26 ppm compared to 0.37 ppm, p < 0.001). The lower appointment rate in older patients contrasts with previous studies which have found older patients have higher utilisation rates [39], and may be a reflection of the particular characteristics of this patient cohort. Late diagnosis has been reported in the literature to be associated with sustained increased healthcare costs [40], in this study there was some evidence that those diagnosed late had higher mean outpatient costs (Table 4), however the differences were not significant using multivariate analysis.

There are several limitations to this study. The data collection was restricted to one year, so changes over time cannot be examined. The usage and cost of other services (inpatient, emergency, and non-ID outpatient care) were not included in this analysis. While the majority of HIV-related care is now provided on an out-patient basis by the ID team, a small minority of patients use a substantial amount of other services [41]. Newly diagnosed patients with low CD4 counts in particular can have very complex care needs, in this study the inpatient costs of two newly diagnosed patients who were diagnosed late and subsequently died were not captured by this micro-costing study. CUH has a smaller proportion of injecting drug users than the national HIV patient population (5% vs 21% ), however the population served is similar in terms of age, gender, CD4 count and viral load to the national cohort in care [7]. Diagnostic tests were linked to specific outpatient appointments on date and mis-matching may have occurred which could have led to either an under- or over-estimate of the diagnostic testing ordered by the HIV clinic. Private patients were not included in the study. While private patients do not attend the public outpatient clinic so would not generate appointment costs, the health service provider may incur costs as a result of diagnostic tests and/or HAART. The additional cost of obstetric care for HIV patients (i.e. additional to the three consultant visits that antenatal patients are routinely entitled to [42]) was not included in the cost estimates. As the numbers of women attending antenatal care was very small in this study it was not possible to investigate this fully, but to give an indication of the scale of the possible additional costs the average number of antenatal-HIV appointments attended in this study was 3.2 per woman. This study also does not include the cost of medications prescribed to HIV patients other than HAART and prophylactic antibiotics, as these are dispensed outside the hospital setting, while the total cost of vaccines and tuberculin dispensed to the HIV clinic was available but could not be assigned at a patient level. The total estimated cost of vaccines and tuberculin in 2012 was €3,734 (including consumables) and even though this is probably an underestimate as patients may be vaccinated in other healthcare settings, overall it is likely to account for a relatively small proportion of the total costs of ambulatory HIV care in this centre.

## Conclusions

Accurate cost data are essential for ensuring HIV services are effective, efficient, and equitable and cost information should be used to guide policy, planning and implementation [11]. This is particularly pertinent in the current situation in Ireland, as healthcare funding is undergoing restructuring. We have found that HIV outpatient costs were substantially underestimated by routine hospital cost data, and we feel that this information will be vital for the development of realistic setting of HIV outpatient appointment prices in the new funding system. In addition, as demands on the service increase due to greater numbers of patients and more complex care these cost data will be available for cost-effectiveness evaluations of new drugs, technologies and models of care.
